# Effect of ISM1 on the Immune Microenvironment and Epithelial-Mesenchymal Transition in Colorectal Cancer

**DOI:** 10.3389/fcell.2021.681240

**Published:** 2021-07-19

**Authors:** Yuhui Wu, Xiaojing Liang, Junjie Ni, Rongjie Zhao, Shengpeng Shao, Si Lu, Weidong Han, Liangliang Yu

**Affiliations:** ^1^Department of Medical Oncology, Sir Run Run Shaw Hospital, College of Medicine, Zhejiang University, Hangzhou, China; ^2^Department of Breast and Thyroid Surgery, Jinhua Municipal Central Hospital, Jinhua, China; ^3^Department of Urinary Surgery, The First People’s Hospital of Fuyang, Hangzhou, China; ^4^Institute of Translational Medicine, Zhejiang University, Hangzhou, China; ^5^Department of Gastroenterology, Sir Run Run Shaw Hospital, College of Medicine, Zhejiang University, Hangzhou, China

**Keywords:** colorectal cancer, ISM1, EMT, immunosuppressive, microenvironment

## Abstract

**Background:** An increasing number of studies have shown that Isthmin 1 (ISM1), a secreted protein, is important in tumorigenesis and invasion, including in colorectal cancer (CRC). However, the mechanisms are still unclear. This study aims to explore the function and prognosis capacity of ISM1 in CRC.

**Methods:** We investigated the expression of ISM1 in 18 CRC tissues vs. adjacent normal tissues from GSE50760, 473 CRC tissues vs. 41 normal tissues from The Cancer Genome Atlas (TCGA), and across gastrointestinal cancer types. Differences were further confirmed in CRC tissues via quantitative real-time polymerase chain reaction (qRT-PCR). Then, we analyzed correlations between clinicopathologic features and ISM1 expression, including prognostic prediction value, using the Kaplan–Meier method and multivariate Cox regression. Gene set enrichment analysis (GSEA) was performed to identify ISM1-related pathways. *In vitro* experiments were performed to verify the role of ISM1 in epithelial-mesenchymal transition (EMT) and CRC progression.

**Results:** Multiple datasets showed that ISM1 is upregulated in CRC tissues, which was validated. Patients with higher ISM1 expression had shorter overall survival (OS), and ISM1 expression served as an independent prognostic factor. Enrichment analysis showed that ISM1 upregulation was positively correlated with cancer-related pathways, such as EMT, hypoxia, and the Notch and KRAS signaling pathways. We were exclusively interested in the connection between ISM1 and EMT because 71% of genes in this pathway were significantly positively co-expressed with ISM1, which may account for why patients with higher ISM1 expression are prone to regional lymph node involvement and progression to advanced stages. In addition, we found that ISM1 was positively correlated with multiple immunosuppressive pathways such as IL2/STAT5, TNF-α/NF-κB, and TGF-β, and immune checkpoints, including PD-L1, PD-1, CTLA-4, and LAG3, which may account for upregulation of ISM1 in immunotherapy-resistant patients. Notably, through *in vitro* experiments, we found that ISM1 promoted EMT and colon cancer cell migration and proliferation.

**Conclusion:** ISM1 is critical for CRC development and progression, which enhances our understanding of the low response rate of CRC to immunotherapy via immunosuppressive signaling pathways.

## Introduction

Colorectal cancer (CRC) is one of the most common malignant tumors worldwide and the third leading cause of global cancer-related mortality (Bray et al., 2018). Great advancements in clinical diagnosis and comprehensive treatment have partially prolonged survival time, but the prognosis of advanced patients is still very poor (Piawah and Venook, 2019; Sveen et al., 2020). Distant invasion and metastasis are responsible for up to 90% of CRC-related deaths (Arnold et al., 2017). Therefore, searching for reliable biomarkers for early diagnosis is warranted.

Isthmin 1 (ISM1) was a secreted protein, and is important for embryonic and postnatal development (Osório et al., 2014). In recent years, growing evidence has shown that aberrant expression of ISM1 can affect the biological behavior of cancer. For example, ISM1 upregulation mediated by lncRNA H19 enhances carcinogenesis and metastasis of gastric cancer (Li et al., 2014) and can also promote cell proliferation and migration in hepatocellular cancer, regulated by hsa_circ_0091570/miR-1307 (Wang et al., 2019). There has also been a research in CRC. Zheng et al. (2019) reported increased expression of ISM1 in CRC cell lines compared with normal cells and that ISM1 participates in the process of cell proliferation and apoptosis regulated by miR-1307-3p. However, each gene may perform its biological function in a complex regulatory network, and whether there are other mechanisms of ISM1 in CRC deserves further research. In addition, Valle-Rios et al. (2014) reported that ISM1 is expressed in mammalian tissues, such as skin, mucosal, and selected lymphocyte populations, but whether it is related to the tumor microenvironment is still unclear.

To clarify the function of ISM1 in CRC, we gathered RNA-seq data of 491 CRC tissues from GEO and TCGA datasets. Moreover, our findings were verified in mouse models and CRC cell lines. This is the first integrative study to characterize ISM1 expression both molecularly and clinically in CRC.

## Materials and Methods

### Data Preparation

We obtained RNA-seq data from GSE50760 (Kim et al., 2014), which contains 18 primary CRC tissues and paired normal tissues, and from TCGA database^1^, which contains 473 CRC tissues and 41 normal tissues in TCGA-COAD cohort. Expression of all genes was normalized in transcripts per million (TPM) format. Then we extracted ISM1 expression data across all samples.

### Differential Expression Analysis of the ISM1 Gene and Its Correlation With Clinicopathological Characteristics

We analyzed ISM1 expression changes between normal tissues and CRC in both the GSE50760 and TCGA cohorts. Then, we obtained an expression overview of ISM1 across gastrointestinal cancer types such as esophageal carcinoma, stomach adenocarcinoma, liver hepatocellular carcinoma, cholangiocarcinoma, pancreatic adenocarcinoma, rectum adenocarcinoma, and CRC in GEPIA database (Tang et al., 2017)^2^. TCGA cohort contained 432 cases of CRC with complete clinical information and a follow-up period of more than 30 days. Correlations between ISM1 and clinicopathological characteristics were further analyzed, including age (<60 vs. ≥60), gender (male vs. female), T stage (T_1–2_ vs. T_3–4_), N stage (N_–_ vs. N_+_), stage (stage I–IV), and prior malignancy (yes vs. no). The overall survival (OS) prediction value of ISM1 was also determined using the “maxstat” package^3^.

### Mouse Models

C57BL/6 mice (male, 8–10 weeks old, Shanghai Institute of Material Medicine, Chinese Academy of Sciences, China) were randomly divided into two groups: groups receiving (A) water only (normal) and (B) DSS for 7 days and water for 14 days (DSS). The induction of CAC was performed according to a previously reported method (Han et al., 2019). First, mice received a single intraperitoneal injection of 10 mg/kg azoxymethane (AOM, MP Biomedicals) and were maintained on a regular diet for 7 days. Then, the mice were continuously fed DSS (2%, MP Biomedicals) for 7 days, followed by a recovery period of 14 days. We cycled the previous steps 3 times. Finally, we sacrificed the mice and removed the colon tumors in preparation for further analysis.

### Cell Lines

NCM460, HCT116, HT29, LoVo, DLD1, SW480, and SW620 cells, were purchased from the Chinese Academy of Sciences (Shanghai, China). NCM460, HCT116, LoVo, SW480, SW620, and DLD1 cells were cultured in RPMI 1640 medium (Gibco, United States); HT29 cells were cultured in DMEM with high glucose (Gibco). All of the above media contained 10% fetal bovine serum (FBS, Gibco). All cell lines were cultured in a 37°C humidified incubator with 5% CO_2_.

### RNA Isolation and Quantification

Total RNA from mouse colon tissues or cell lines was extracted using RNA isolate reagent (#R401-01-AA, Vazyme), according to the manufacturer’s instructions. Then, the RNA was converted to cDNA using the HiScript II 1st Strand cDNA Synthesis Kit (#R312-02, Vazyme). After that, quantitative real-time polymerase chain reaction (qRT-PCR) was performed using MagicSYBR Mixture (#CW3008M, CWBIO). The relative expression of the ISM1 gene was normalized to that of GAPDH. Primer sequence information was as follows: mouse ISM1: forward primer: 5′-GATGGCCCTGACTCCGAAG-3′; reverse primer: 5′-GGTCCCCACTATTTGTCCTGG-3′; human ISM1: forward primer: 5′-CTTCCCCAGACCGCGATTC-3′; reverse primer: 5′-CGACCACCTCTATGGTGACCT-3′.

### Enrichment Analysis of ISM1

To gain further insights into the biological function of ISM1, we calculated the correlation between ISM1 and all other protein-coding genes in 473 CRC tissues from TCGA cohort. Then genes were sorted according to Pearson’s correlation to produce a gene list. Gene set enrichment analysis (GSEA) was performed using the “clusterProfiler” package (Subramanian et al., 2005; Yu et al., 2012). Enriched results with adjusted *P* < 0.05 were considered significant. To understand the activity of a certain pathway in CRC, single sample gene set enrichment analysis (ssGSEA) was performed to calculate the activity score in each tissue based on the gene expression profile (Cui et al., 2020). Since 71% genes in epithelial-mesenchymal transition (EMT) pathway were significantly positively co-expressed with ISM1, we focused on the role of EMT in the development and progression of CRC and made a comparison to EMT activity score in tumor tissues vs. paired normal tissues and lymph node involvement vs. non-involvement.

### Role of ISM1 in an Immunosuppressive Microenvironment

Enrichment results indicated that ISM1 upregulation was significantly positively associated with multiple immunosuppressive pathways. We further investigated correlations between ISM1 and markers of regulatory T cells (CCR8, TGFB1, STAT5B, and FOXP3), M2 macrophages (CD163, VSIG4, and MS4A4A), and T cell exhaustion (TIM-3, CTLA-4, PD-1, and LAG3) and PD-L1.

To further confirm the suppressive role of ISM1 in immunotherapy, we obtained RNA-seq data and response information from PD-L1 mAb-treated metastatic urothelial cancer patients from IMvigor210 (Mariathasan et al., 2018), which contained 61 complete response (CR)/partial response (PR), and 183 stable disease (SD)/progressive disease (PD) cases. And these patients consisted of three distinct immunological phenotypes: 62 cases of immune inflamed, 113 cases of immune excluded, and 69 cases of immune desert. We compared the expression of ISM1 in patients with different PD-L1 response and immunological phenotypes.

### RNA Interference, Plasmid Construction and Transfections

Cells were transfected with siRNA using RNAiMAX (Thermo Fisher Scientific) according to the manufacturer’s protocol. siRNAs were purchased from RiboBio (Guangzhou, China), and the target sequences were as follows: si-ISM1-1: 5′–GGCAGAATCCAAATATCCA–3′; si-ISM1-2: 5′–GCAAAAGC GAGTTCTTAAA–3′; and si-ISM1-3: 5′–GACACCACA TCAGAAACCA–3′.

Cells were transfected with plasmids using jetPRIME (Polyplus, United States) according to the manufacturer’s protocol. The plasmids used in this study were purchased from GENERAY Biotechnology (Shanghai, China).

### Immunoblot Analysis

Proteins in cell lysates were separated via sodium dodecyl sulfate-polyacrylamide gel electrophoresis and transferred to PVDF membranes (Millipore, Bedford, MA, United States). Membranes were blocked with 5% milk in TBST buffer [10 mM Tris–Cl (pH 7.4), 150 mM NaCl, 0.1% Tween 20] at room temperature and incubated overnight at 4°C with the indicated primary antibodies. Then, membranes were incubated with corresponding secondary antibodies at room temperature for 1 h, and proteins were detected with enhanced chemiluminescence reagent (SuperSignal Western Pico Chemiluminescent Substrate; Pierce, United States). Antibodies against the following proteins were used in this study: β-actin (Cell Signaling Technology, CST, United States); ISM1 (Abcam, United States); N-cadherin (CST, United States); E-cadherin (CST, United States); and Snail (CST, United States); ZEB1 (CST, United States).

### Transwell Assay

Cells were harvested and resuspended at 1 × 10^6^ cells/mL (LoVo, DLD1) or 5 × 10^5^ cells/mL (HT29) in FBS-free culture medium. Then, 600 μL of culture medium containing 10% FBS was added to the lower chambers and 200 μL of cell suspension was added to the upper chambers containing the cell culture inserts (Corning, United States). After 24 h, cells on the top surface of the inserts were removed with a cotton swab. Cells on the bottom surface of the inserts were fixed with 4% paraformaldehyde for 5 min at room temperature and then washed with PBS. Migrated cells were stained with DAPI for 10 min and then washed with PBS. Migrated cells were visualized and photographed.

### Cell Counting Kit-8 (CCK8) Assay

The effect of ISM1 expression on human colon cancer cell lines was analyzed using the CCK8 (Meilunbio, Shanghai, China) assay. A 200 μL cell suspension containing 5,000 cells was seeded in each well (96-well plate). At different time points (0, 24, 48, and 72 h), the supernatants were removed, and 100 μL CCK8 solution (10 μL CCK8: 90 μL medium) was added to each well and incubated with the cells for 3 h before analysis. The absorbance at 450 nm was measured.

### Statistical Analysis

Differences in ISM1 expression in CRC vs. normal tissues was calculated with a Mann–Whitney test in GraphPad (version 7.00). The role of ISM1 in clinicopathological characteristics and PD-L1 mAb response was calculated with a Wilcoxon test or Kruskal–Wallis test in R software (version 3.5.0). Overall survival rates were calculated using Kaplan–Meier survival curves and a log-rank test. Cox regression was used to determine hazard ratios (HRs) and to identify independent prognostic factors. Connections between ISM1 expression and other genes were determined using Pearson’s correlation. EMT activity score differences across cancer types were calculated with paired Wilcoxon tests in R software (version 3.5.0). The EMT activity score difference between lymph node involvement and non-involvement was calculated using a Mann–Whitney test in GraphPad (version 7.00). qRT-PCR data are expressed as the mean ± standard error, and statistical significance was determined with Student’s *t* test. *P* < 0.05 was considered statistically significant.

## Results

### ISM1 Is Upregulated in CRC vs. Normal Tissues

**FIGURE 1:**
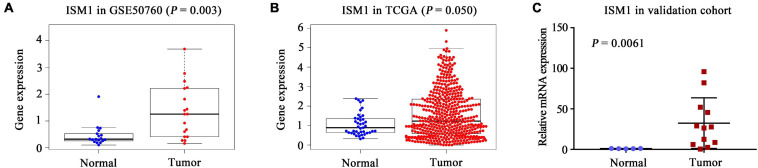
Landscape of ISM1 expression changes. Analysis of differential ISM1 expression between CRC tissues and normal tissues was performed in the **(A)** GSE50760 dataset, **(B)** TCGA dataset, and **(C)** validation cohort.

In both the GSE50760 and TCGA datasets, the expression of ISM1 was significantly higher in CRC tissues than in normal tissues (*P* = 0.003, 0.050; Figures 1A,B). To validate the ISM1 expression changes, mice CRC was induced using AOM and DSS. Then we extracted RNA from CRC or normal colon tissues and performed qRT-PCR and found that ISM1 was consistently significantly higher in CRC tissues than in normal colon tissues (*P* = 0.0061; Figure 1C). From the expression landscape across gastrointestinal cancer types, ISM1 showed a higher expression trend in esophageal carcinoma and rectal adenocarcinoma but a lower expression trend in stomach adenocarcinoma, liver hepatocellular carcinoma, cholangiocarcinoma, and pancreatic adenocarcinoma (*P* > 0.05; Supplementary Figure 1).

### ISM1 Is Correlated With Adverse Clinicopathological Characteristics in CRC

**FIGURE 2:**
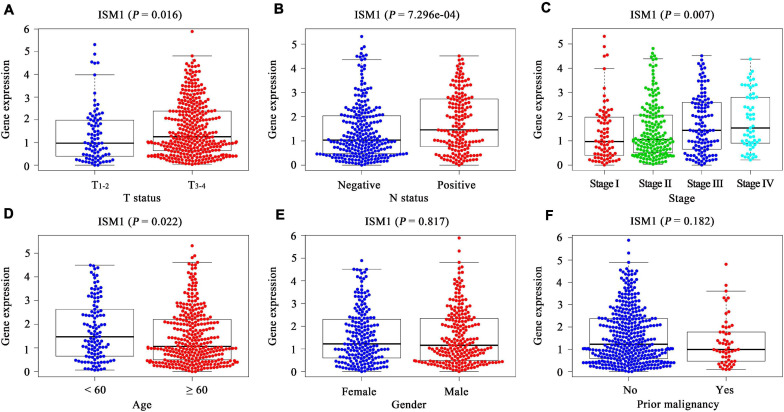
Correlation between clinicopathological characteristics and ISM1 in CRC. **(A–F)** ISM1 expression changes in patients with T_3–4_ (vs. T_1–2_), lymph node involvement, advanced stages, age <60 (vs. ≥60), and prior malignancy (yes vs. no). *P* < 0.05 is statistically significant.

In TCGA cohort, after comparing the expression of ISM1 among cases with different clinicopathological characteristics, we found that expression was higher in patients with age < 60 (*P* < 0.05), a larger tumor size (*P* < 0.05), lymph node involvement (*P* < 0.001), and advanced stages (*P* < 0.01) (Figures 2A–D). These differences indicate that ISM1 upregulation is associated with aggressive behavior in CRC patients. Differences were not found in gender or prior malignancy (Figures 2E,F).

### ISM1 Predicts Shorter OS in CRC

**FIGURE 3:**
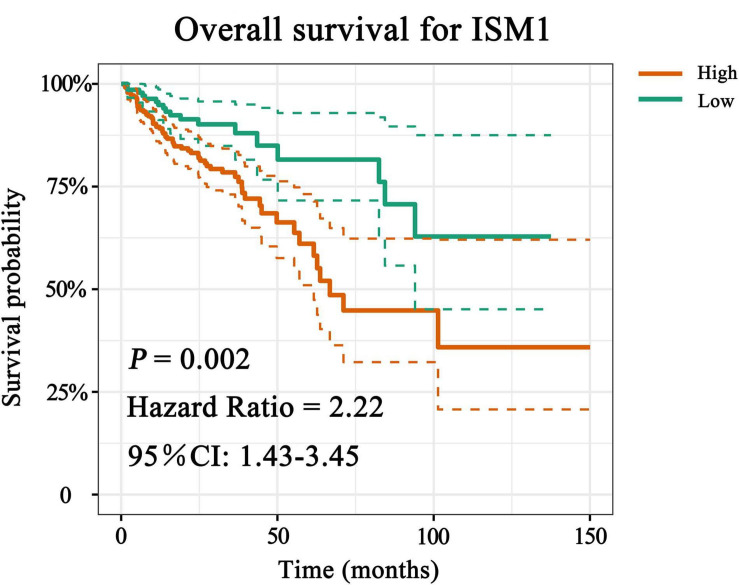
Overall survival (OS) analysis of ISM1. The expression of ISM1 was significantly related to OS in TCGA-CRC cohort. Yellowish brown indicates high ISM1 expression, while dark green indicates low expression. Dotted lines represent confidence intervals. The horizontal axis indicates OS time (months), and the vertical axis is survival probability. *P* < 0.05 is statistically significant.

With the method of maximally selected rank statistics, we identified the most effective ISM1 expression cutoff to distinguish CRC prognosis in TCGA cohort (*P* = 0.002, HR = 2.22, 95% CI: 1.43–3.45; Figure 3). From the results of Kaplan–Meier curves and Cox regression, higher ISM1 expression indicated a shorter OS. Multivariate analysis showed that ISM1, tumor size and lymph node involvement were all independent prognostic factors (Table 1). In this way, ISM1-related aggressive biological behavior may account for its negative effect on OS. Clinically, the prognosis of patients with different clinicopathological features varies greatly, and the treatment they receive is not exactly the same. We further conducted a subgroup analysis based on age, sex, T status, lymph node status, stage, and malignancy history. The results showed that ISM1 overexpression had a better performance in CRC patients with the following characteristics: ≥60 (years), male, T_3–4_, negative lymph nodes, and prior malignancy, and all of these patients exhibited a shorter OS (Supplementary Figure 2). Interestingly, the expression of ISM1 was higher in advanced CRC (Figure 2C). However, we found that there was only a slight relationship between ISM1 and OS in the stage II–III subgroups. ISM1 was not related to the prognosis of patients in stage I/IV, which may be caused by the lower distribution of cases in these subgroups.

**TABLE 1:** Univariate and multivariate analysis of clinicopathological characteristics and ISM1 with overall survival in TCGA-COAD cohort.

**TCGA-COAD cohort (*n*=432)**	**Univariate analysis**		**Multivariate analysis**	
	HR (95% CI)	*P*	HR (95% CI)	*P*
**Age (vs. <60)**	1.303 (0.781–2.175)	0.311		
**Gender (vs. female)**	1.133 (0.736–1.743)	0.571		
**T status (vs. T_1–2_)**	4.275 (1.562–11.701)	0.005	2.818 (1.005–7.903)	0.049
**N status (vs. N_0_)**	2.813 (1.805–4.384)	<0.001	2.247 (1.423–3.547)	<0.001
**Prior malignancy (vs. yes)**	0.612 (0.355–1.055)	0.077		
**ISM1 (vs. low)**	2.259 (1.334–3.824)	0.002	1.94 (1.140–3.300)	0.014

### ISM1 Is Correlated With Multiple Cancer-Related Pathways

**FIGURE 4:**
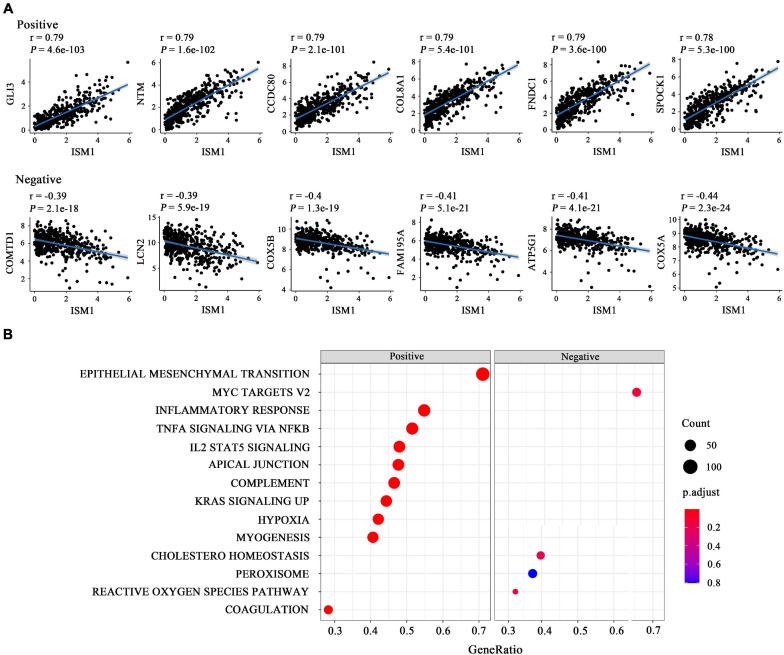
Functional enrichment analysis of ISM1 in CRC. **(A)** Correlation between ISM1 and the remaining 19505 genes. The top six positively and negatively correlated genes are shown. **(B)** GSEA of the 19505 genes was performed using Pearson’s correlation, and the top 10 positively and negatively correlated pathways are shown. Pathways in the left box were found to be positively correlated with ISM1, while those in the right box were found to be negative correlated with ISM1. The color of the dots varies from blue to red, representing the significance of changes: red indicates statistical significance. When many genes were found to be correlated with ISM1 in a pathway, the size of the dot larger. The horizontal axis represents the ratio of correlated genes among all the genes in each pathway. *P* < 0.05 is statistically significant.

The results mentioned above showed that ISM1 is associated with CRC development and progression. We then performed GSEA to determine ISM1-related biological functions through GSEA based on the Pearson’s correlation value for 19505 identified genes. The top six correlated genes (positive and negative) were displayed in the form of a scatter plot (Figure 4A). For example, ISM1 showed a positive correlation with GLI3 (cor = 0.79, *P* = 4.6e-103), CCDC80 (cor = 0.79, *P* = 2.1e-101), COL8A1 (cor = 0.79, *P* = 5.4e-101), FNDC1 (cor = 0.79, *P* = 3.6e-100), and SPOCK1 (cor = 0.78, *P* = 5.3e-100) but a negative correlation with COMTD1 (cor = -0.39, *P* = 2.1e-18), LCN2 (cor = -0.39, *P* = 5.9e-19), COX5B (cor = -0.4, *P* = 1.3e-19), FAM195A (cor = -0.41, *P* = 5.1e-21), ATP5G1 (cor = -0.41, *P* = 4.1e-21), and COX5A (cor = -0.44, *P* = 2.3e-24).

**FIGURE 5:**
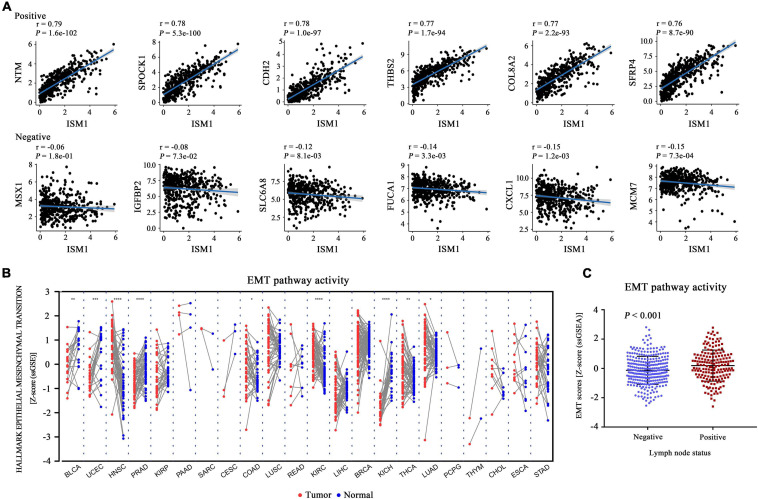
Epithelial-mesenchymal transition (EMT) activity score in all cancer types. **(A)** Pearson’s correlation between ISM1 and genes in the EMT pathway was calculated, and the top six positively and negatively correlated genes are shown. **(B)** The pathway activity score for EMT was calculated and compared across 22 cancers from TCGA between tumor tissues (red dot) and paired normal tissues (blue dot). **(C)** EMT activity score for 300 lymph node non-involvement tissues (41 normal tissues and 259 negative lymph node tissues) and 173 lymph node involvement tissues. *P < 0.05; **P < 0.01; ***P < 0.001.

The GSEA results showed that multiple cancer-related pathways were significantly associated with ISM1, including EMT, hypoxia, the KRAS signaling pathway, angiogenesis, the Notch signaling pathway, and the Hedgehog signaling pathway (Figure 4B and Supplementary Figure 3). Among them, we exclusively focused on the correlation between ISM1 and EMT because 71% of genes in this pathway were significantly positively co-expressed with ISM1. The top six positively correlated genes (NTM, SPOCK1, CDH2, THBS2, COL8A2, and SFRP4, cor > 0.75, *P* < 0.001) and negatively correlated genes (MSX1, IGFBP2, SLC6A8, FUCA1, CXCL1, and MCM7, | cor| : 0.06–0.15, *P:* 0.00073–0.18) in EMT are displayed in Figure 5A. Additionally, the EMT activity score was notably increased in CRC vs. paired normal tissues (Figure 5B and Supplementary Table 1) and in lymph node involvement vs. non-involvement tissues (Figure 5C), indicating that the relationship between ISM1 and EMT is vital in CRC progression.

### ISM1 Is Correlated With the Immunosuppressive Microenvironment in CRC

**FIGURE 6:**
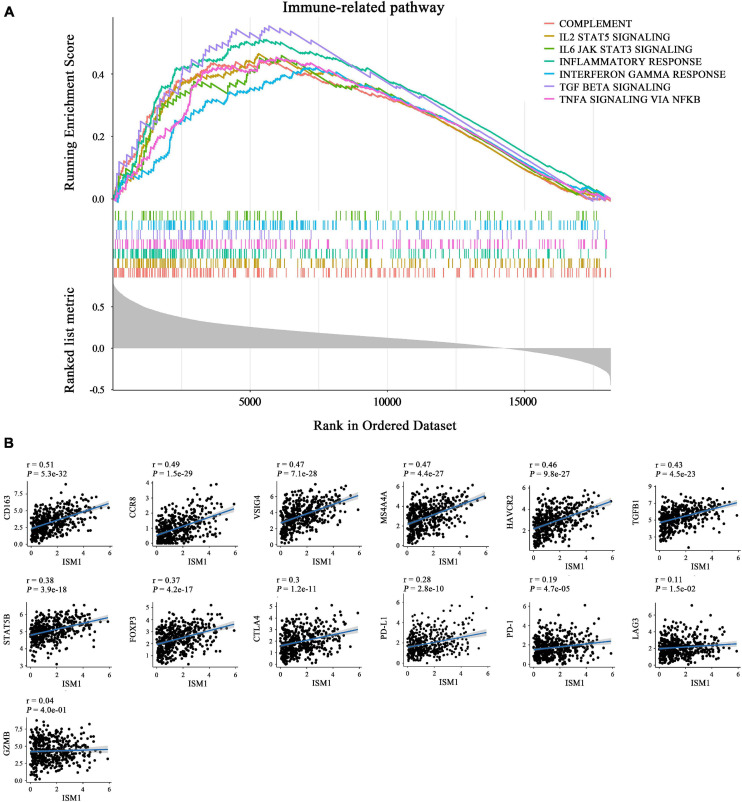
Role of ISM1 in an immunosuppressive microenvironment. **(A)** Seven significantly enriched ISM1-related immunosuppressive pathways in CRC. The short vertical lines in the middle of the figure represent the distribution of ISM1-related genes among the 19505 genes. **(B)** Correlation between ISM1 and immunosuppressive markers of M2 macrophages (CD163, VSIG4, and MS4A4A), Tregs (CCR8, TGFB1, STAT5B, and FOXP3), and T cell exhaustion (TIM-3, CTLA-4, PD-1, and LAG3) and PD-L1. Correlation strength is defined as follows: “0.00–0.29” indicates weak; “0.30–0.59” indicates moderate; “0.60–1.0” indicates strong. *P* < 0.05 is statistically significant.

The GSEA results also showed that ISM1 was highly associated with immune-related pathways, such as the IL2/STAT5, TNF-α/NF-κB, TGF-β, IFN-γ, and IL6/JAK/STAT3 signaling pathways (Figure 6A and Supplementary Figure 4). Previous studies have demonstrated that most of these pathways are important to induce strong suppression of the antitumor immune response through Treg cell infiltration (Shi et al., 2018), CD8^+^ T cell exhaustion (Liu et al., 2021), and stabilization of PD-L1 (Lim et al., 2016). However, corresponding reports on ISM1 in immunosuppression are rare. For further understanding, we calculated correlations between ISM1 and immunosuppressive markers. Notably, most correlations were highly significant, including correlations with M2 macrophages (CD163, VSIG4, and MS4A4A, cor: 0.47–0.51, *P* < 0.001) and Tregs (CCR8, TGFB1, STAT5B, and FOXP3, cor: 0.37–0.48, *P* < 0.001). ISM1 showed a weak to moderate positive correlation with the expression of exhausted T cell markers, such as TIM-3, (cor = 0.46, *P* < 0.001), CTLA-4 (cor = 0.30, *P* < 0.001), PD-1 (cor = 0.19, *P* < 0.001), and LAG3 (cor = 0.11, *P* < 0.01). The correlation between PD-L1 and ISM1 was 0.28 (*P* < 0.001) (Figure 6B).

Moreover, we analyzed the role of ISM1 in microenvironment in patients treated with PD-L1 mAb, and found it was overexpressed in immune-excluded tumors vs. inflamed (Supplementary Figure 5A), and SD/PD patients with immune-excluded tumors vs. CR/PR patients with inflamed tumors (Supplementary Figure 5B), indicating that ISM1 may participate in the exclusion of CD8 + T cells entering the parenchyma, which was associated with the activation of TGF-β signaling pathway (Mariathasan et al., 2018). Notably, it supported our previous findings that CRC tissues with overexpressed ISM1 showed a higher activity score of TGF-β signaling pathway (Supplementary Figure 4A). We may speculate from these results that high ISM1 expression may create an immunosuppressive microenvironment to drive immune escape and induce resistance to immunotherapy.

### *In vitro* Experiments Confirmed That ISM1 Promotes EMT and CRC Progression

**FIGURE 7:**
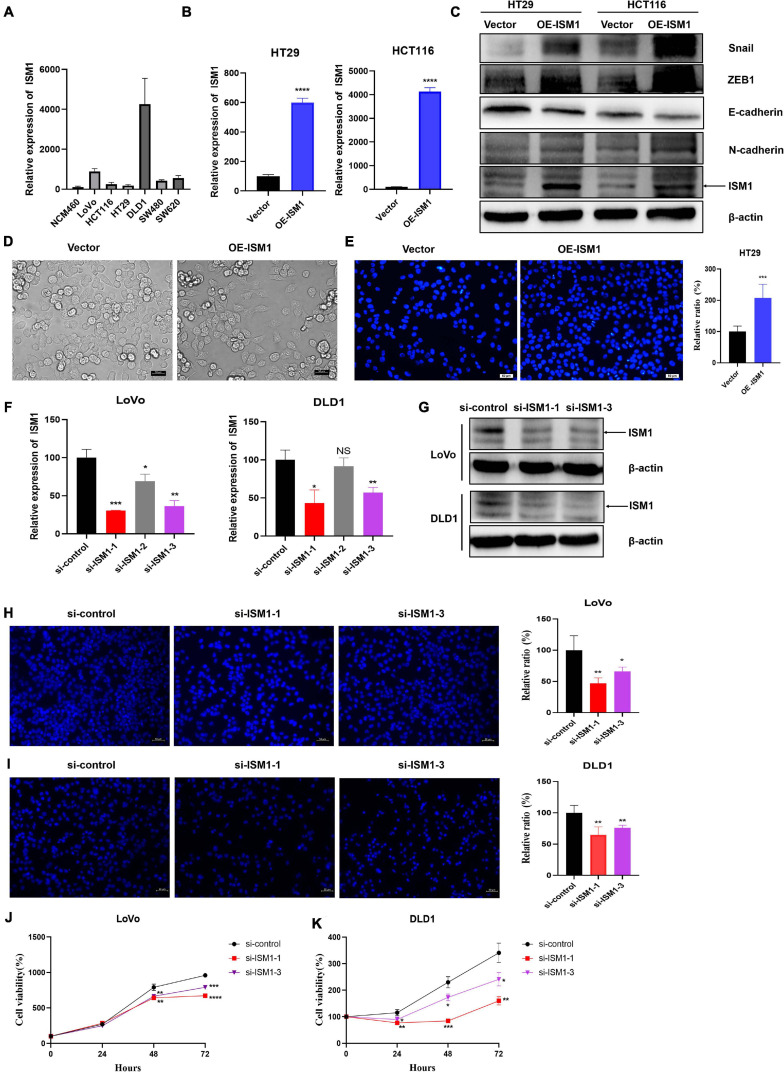
ISM1 promotes EMT and CRC progression *in vitro*. **(A)** Quantitative reverse transcription polymerase chain reaction (qRT-PCR) analysis of ISM1 mRNA levels in NCM460, LoVo, HCT116, HT29, DLD1, SW480, and SW620 cells (*n* = 3, mean ± SD). **(B)** The overexpression (OE) efficiency of ISM1 plasmid (OE-ISM1) in HT29 and HCT116 cells was analyzed via qRT-PCR. Student’s *t* test; ****P < 0.0001; *n* = 3, mean ± SD. **(C)** Immunoblot analysis of ISM1, N-cadherin, E-cadherin, Snail, and ZEB1 in HT29 and HCT116 cells transfected with Vector or OE-ISM1 plasmid. **(D)** Phase contrast images of HT29 cells transfected with Vector or OE-ISM1 plasmid. Scale bars: 50 μm. **(E)** Transwell assay of HT29 cells transfected with Vector or OE-ISM1 plasmid. Student’s *t* test; **P < 0.01; *n* = 5, mean ± SD. Scale bars: 50 μm. **(F)** The knockdown efficiency of si-ISM1-1, si-ISM1-2 and si-ISM1-3 in LoVo and DLD1 cells was analyzed via qRT-PCR. Student’s *t* test; *P < 0.05, **P < 0.01, ***P < 0.001, NS, no significance; *n* = 3, mean ± SD. **(G)** The knockdown efficiency of si-ISM1-1 and si-ISM1-3 in LoVo and DLD1 cells was analyzed via immunoblot experiments. **(H)** Transwell assay of LoVo cells transfected with si-control, si-ISM1-1, or si-ISM1-3. Student’s *t* test; *P < 0.05, **P < 0.01; *n* = 5, mean ± SD. Scale bars: 50 μm. **(I)** Transwell assay of DLD1 cells transfected with si-control, si-ISM1-1, or si-ISM1-3. Student’s *t* test; **P < 0.01; *n* = 5, mean ± SD. Scale bars: 50 μm. **(J)** Proliferation of LoVo cells transfected with si-control, si-ISM1-1, or si-ISM1-3 was determined using CCK-8 assays. **(K)** Proliferation of DLD1 cells transfected with si-control, si-ISM1-1, or si-ISM1-3 was determined using CCK-8 assays.

To confirm the role of ISM1 in EMT and CRC progression, *in vitro* experiments were performed. We first detected ISM1 expression levels in a human normal colon epithelial cell line (NCM460) and in colon cancer cell lines (LoVo, HCT116, HT29, DLD1, SW480, and SW620). As shown in Figure 7A, the ISM1 level in colon cancer cell lines was higher than that in the normal colon epithelial cell line, which was consistent with previous results showing that the ISM1 expression level is higher in CRC tissues than in normal tissues (Figures 1A–C). HT29 and HCT116 cells exhibited lower ISM1 expression, and DLD1 and LoVo cells exhibited higher ISM1 expression. EMT is usually monitored by assessing the protein levels of N-cadherin (mesenchymal marker), E-cadherin (epithelial marker), and the EMT-inducing transcription factors (EMT-TFs) Snail and ZEB (Singh et al., 2018). Therefore, we overexpressed ISM1 in HT29 and HCT116 cells and verified the overexpression (OE) efficiency by qRT-PCR analysis (Figure 7B). Then, we detected these important EMT-associated molecules by immunoblot experiments. As shown in Figure 7C, ISM1 OE significantly decreased the level of the epithelial marker E-cadherin but increased the levels of the mesenchymal marker N-cadherin and the EMT-inducing transcription factor Snail and ZEB1. We also found that ISM1-overexpressed HT29 cells had an obvious change of cellular morphology (Figure 7D). These results suggest that ISM1 promotes EMT.

To further examine the biological function of ISM1 in CRC, we analyzed cell migration ability of HT29 cells transfected with Vector or OE-ISM1 plasmid. As shown in Figure 7E, ISM1 OE significantly elevated HT29 cells migration ability, with increase of 1.5-fold. We then generated three small interfering RNAs (siRNAs), namely, si-ISM1-1, si-ISM1-2, and si-ISM1-3. si-ISM1-1 exhibited the highest knockdown (KD) efficiency, followed by si-ISM1-3 and si-ISM1-2 (Figure 7F). KD efficiency was further determined by immunoblot experiments (Figure 7G). We then employed si-ISM1-1 and si-ISM1-3 in the ISM1-high colon cancer cell lines LoVo and DLD1. As shown in Figures 7H,I, ISM1 KD reduced the migration of LoVo cells by 53.1% (si-ISM1-1, *P* < 0.01) and 33.8% (si-ISM1-3, *P* < 0.05) and of DLD1 cells by 35.1% (si-ISM1-1, *P* < 0.01) and 23.8% (si-ISM1-3, *P* < 0.01) compared with control siRNA (si-control)-transfected cells. We also found that ISM1 KD significantly impaired the proliferation ability of colon cancer cells (Figures 7J,K). Thus, our data demonstrate that ISM1 promotes EMT and CRC progression.

## Discussion

In our study, we found that the expression of ISM1 was upregulated in CRC tissue (vs. normal) and in patients <60 years of age (vs. those ≥60), T_3–4_ (vs. T_1–2_), lymph node involvement, and advanced stages and served as an independent OS prediction factor. Enrichment analysis showed that multiple cancer-related pathways and immune-related pathways were closely associated with ISM1.

Previous studies reported that ISM1 is upregulated in CRC cell lines, which is consistent with our results, and participates in the process of cell proliferation and apoptosis regulated by miR-1307-3p (Shi et al., 2018). However, we found that there were more important mechanisms of ISM1 in CRC that have not been reported. In the GSEA results, enriched pathways, such as the EMT, hypoxia, KRAS signaling, angiogenesis, Notch and Hedgehog signaling pathways, were significantly positively associated with ISM1, and most of these pathways are vital to cell proliferation, migration and resistance in cancer (Wang et al., 2020; Andrysik et al., 2021; Cai et al., 2021), including CRC. We exclusively focused on the EMT signaling pathway because 71% of genes in the pathway exhibited a significantly positive correlation with ISM1. Additionally, genes that were highly correlated with ISM1 (cor > 0.7) but not members of the EMT pathway, such as ITGBL1 (Matsuyama et al., 2019), NRP-2 (Liu et al., 2019; Zhang et al., 2019), NOX4 (Shen et al., 2020), and GLI3 (Shen et al., 2021), have already been demonstrated to promote EMT, cell invasion and migration in CRC. EMT is a process during which epithelial cells lose epithelial features and acquire mesenchymal phenotypes and behavior (Yang et al., 2020). EMT is a key process in tumor metastasis, affecting various malignancies (Bai et al., 2021). Cancer cells with EMT also acquire resistance to some chemotherapy. For example, EMT-TFs (ZEB1, SNIAL, SLUG) have been demonstrated to confer resistance to oxaliplatin and cisplatin in breast, ovarian, colon, and pancreatic cancers (Guo et al., 2012; Lim et al., 2013). Interestingly, EMT in cancer cells gives rise to a population of cells with stem-like properties, namely, cancer stem cells (CSCs), which may persist after chemotherapeutic treatment and lead to tumor relapse (Mani et al., 2008; Morel et al., 2008; Shibue and Weinberg, 2017). Additionally, EMT can also modulate immune response. Kudo-Saito et al. (2009) reported that EMT in melanoma induce regulatory T cells and impaired dendritic cells, which leads to resistance to immunotherapy. Therefore, we speculate that EMT induced by ISM1 upregulation played an important role in regional lymph node involvement and advanced stages of CRC. Indeed, our *in vitro* experiments showed that ISM1 could promote EMT. Moreover, ISM1 KD significantly impaired the migration and proliferation abilities of colon cancer cells.

From the assessment of enriched immune-related pathways, including IL2/STAT5, TNF-α/NF-κB, TGF-β, IFN-γ response, and IL6/JAK/STAT3, ISM1 was predicted to be vital in creating an inhibitory immune microenvironment. Shi et al. (2018) found that IL-2/STAT5 was important for maintaining Treg cell homeostasis and migration ability and regulating CD8^+^ T cell exhaustion (Liu et al., 2021). Owing to the decreased number of intratumoral Tregs, the combination of endoglin antibodies and PD-1 inhibition induced complete regression in 30–40% of CRC mice, producing durable tumor responses (Schoonderwoerd et al., 2020). A high proportion of Treg cells was found to induce resistance to anti-CTLA-4 blocking mAbs in patients with advanced cancer (Coutzac et al., 2020), but such suppression of immunotherapy could be reversed by the TGF-β pathway inhibitor galunisertib (Holmgaard et al., 2018). The TNF-α/NF-κB signaling pathway is a major pathway that stabilizes programmed cell death-ligand 1 (PD-L1) via COP9 signalosome 5 (CSN5), driving cancer cell immunosuppression against T cell surveillance (Lim et al., 2016). The JAK/STAT3 signaling pathway can promote the expression of PD-L1 in CRC (Li et al., 2019). The correlations between ISM1 and markers of suppressive immune cells (M2 macrophages, Tregs, T cell exhaustion) and the overexpression of ISM1 detected in PD1-resistant patients further confirmed our speculation that ISM1 upregulation is potentially involved in immunotherapy resistance via immune-related pathways in CRC.

As mentioned above, research on ISM1 in cancer is still in its infancy. The limitations of this study were also accompanied by advantages. This is the first study to find that ISM1 is associated with multiple important cancer-related pathways, especially EMT, and multiple important immunosuppressive signaling pathways, which provides us with evidence supporting application of ISM1 inhibitors in CRC immunotherapy. However, the mechanisms underlying ISM1 promotion of CRC progression need to be further studied.

In summary, ISM1 plays an important role in CRC development and progression via several pathways, such as EMT. Furthermore, investigating ISM1 and immunosuppressive signaling pathways enhances our understanding of the low response rate of CRC to immunotherapy.

## Data Availability Statement

The datasets presented in this study can be found in online repositories. The names of the repository/repositories and accession number(s) can be found in the article/Supplementary Material.

## Ethics Statement

The animal study was reviewed and approved by the Experimental Animal Welfare Ethics Review Committee of Zhejiang University.

## Author Contributions

WH and LY: conceptualization and supervision. YW, XL, and JN: investigation. RZ, YW, SS, XL, and SL: analysis. YW, JN, and RZ: wrote original draft. WH: review, editing, and final approval. All authors contributed to the article and approved the submitted version.

## Conflict of Interest

The authors declare that the research was conducted in the absence of any commercial or financial relationships that could be construed as a potential conflict of interest.

## Footnotes

1 https://portal.gdc.cancer.gov/

2 http://gepia.cancer-pku.cn/detail.php

3 https://CRAN.R-project.org/package=maxstat

## Supplementary Material

Supplementary Figure 1 Expression of ISM1 in gastrointestinal cancers. ESCA, esophageal carcinoma; STAD, stomach adenocarcinoma; LIHC, liver hepatocellular carcinoma; CHOL, cholangiocarcinoma; PAAD, pancreatic adenocarcinoma; COAD, colon adenocarcinoma; READ, rectum adenocarcinoma. ^∗^*P* < 0.05.

Supplementary Figure 2 Subgroup OS analysis of ISM1 expression in CRC. Patients were stratified based on age **(A)**, sex **(B)**, T status **(C)**, lymph node status **(D)**, stage **(E)**, and malignancy history **(F)**. *P* < 0.05 is statistically significant.

Supplementary Figure 3 Activity score of multiple cancer-related pathways associated with ISM1 expression. **(A–F)** Epithelial mesenchymal transition, angiogenesis, the Hedgehog signaling pathway, the KRAS signaling pathway and hypoxia showed higher activity scores in CRC patients overexpressing ISM1.

Supplementary Figure 4 Activity score of multiple immune-related pathways associated with ISM1 expression. **(A–F)** TGF-β, inflammatory response, IL2/STAT5, TNF-α/NF-κB, IFN-γ response, and the IL6/JAK/STAT3 signaling pathway showed higher activity scores in CRC patients overexpressing ISM1.

Supplementary Figure 5 Expression of ISM1 in microenvironment in patients treated with immunotherapy. **(A)** Differentially expressed analysis of ISM1 in three distinct immunological phenotypes, **(B)** SD/PD patients with immune-excluded tumors vs. CR/PR patients with inflamed tumors. Complete response (CR), partial response (PR), and progressive disease (PD). *P* < 0.05 is statistically significant.

Supplementary Table 1 Information for calculating EMT activity score across 22 cancers from TCGA.
