# Supplementary figures and images for: Effect of ISM1 on the Immune Microenvironment and Epithelial-Mesenchymal Transition in Colorectal Cancer
Source: Front Cell Dev Biol. 2021 Jul 19;9:681240. doi: 10.3389/fcell.2021.681240 (PMC8326811; doi:10.3389/fcell.2021.681240)

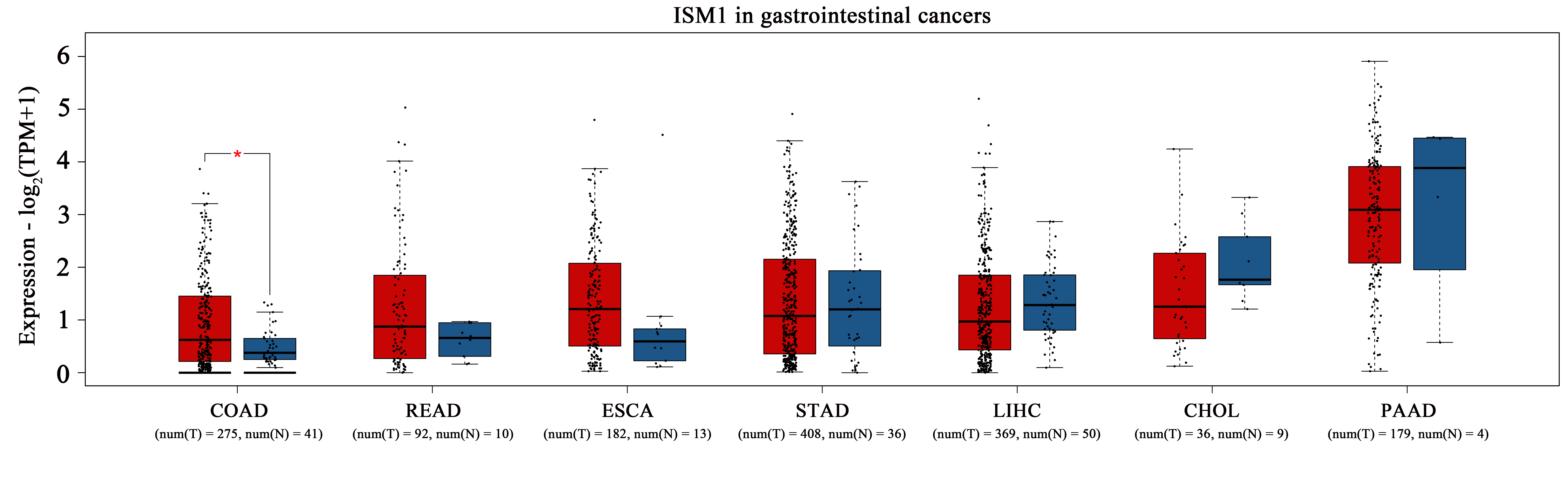

Supplement: Supplementary Figure 1 — Expression of ISM1 in gastrointestinal cancers. ESCA, esophageal carcinoma; STAD, stomach adenocarcinoma; LIHC, liver hepatocellular carcinoma; CHOL, cholangiocarcinoma; PAAD, pancreatic adenocarcinoma; COAD, colon adenocarcinoma; READ, rectum adenocarcinoma. ∗P < 0.05. [file Image_1.TIF]

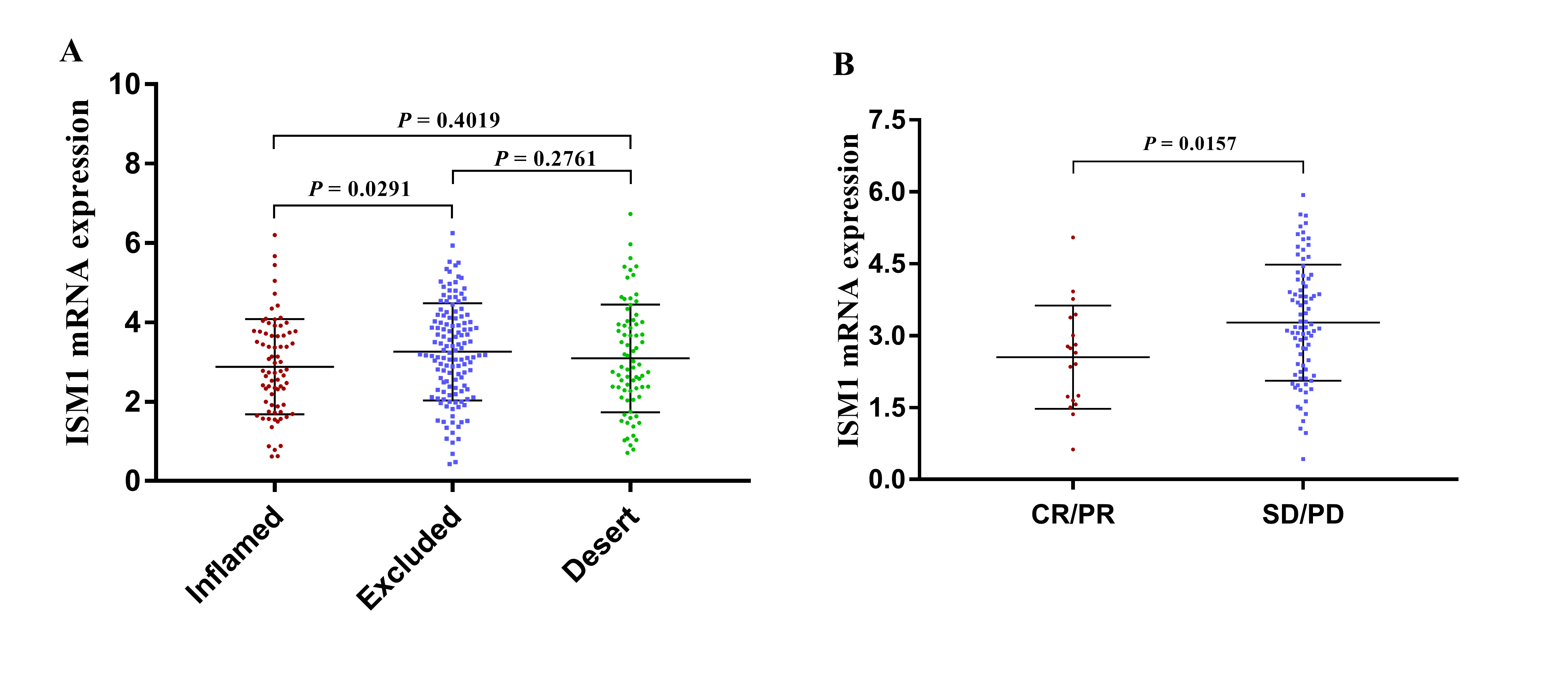

Supplement: Supplementary Figure 5 — Expression of ISM1 in microenvironment in patients treated with immunotherapy. (A) Differentially expressed analysis of ISM1 in three distinct immunological phenotypes, (B) SD/PD patients with immune-excluded tumors vs. CR/PR patients with inflamed tumors. Complete response (CR), partial response (PR), and progressive disease (PD). P < 0.05 is statistically significant. [file Image_5.TIF]
