# Supplementary material for: Effect of ISM1 on the Immune Microenvironment and Epithelial-Mesenchymal Transition in Colorectal Cancer
Source: Front Cell Dev Biol. 2021 Jul 19;9:681240. doi: 10.3389/fcell.2021.681240 (PMC8326811; doi:10.3389/fcell.2021.681240)
Supplement: Supplementary Table 1 — Information for calculating EMT activity score across 22 cancers from TCGA. [file Table_1.pdf]

Supplementary table 1. Information for calculating EMT activity score across 22 cancers from TCGA

Cancer types	Tumor tissues	Adjacent normal tissues	*P*
TCGA-BRCA	98	98	0.421
TCGA-KIRC	71	71	<0.0001
TCGA-LUAD	57	57	0.017
TCGA-THCA	57	57	0.006
TCGA-PRAD	51	51	<0.0001
TCGA-LIHC	50	50	0.132
TCGA-LUSC	50	50	0.629
TCGA-HNSC	43	43	<0.0001
TCGA-COAD	40	40	0.015
TCGA-KIRP	32	32	0.106
TCGA-STAD	32	32	0.139
TCGA-KICH	25	25	<0.0001
TCGA-UCEC	22	22	0.001
TCGA-BLCA	19	19	0.005
TCGA-ESCA	11	11	0.413
TCGA-	9	9	0.129
TCGA-READ	9	9	0.820
TCGA-PAAD	4	4	1.000
TCGA-	3	3	0.250
TCGA-	3	3	0.500
TCGA-SARC	2	2	0.500
TCGA-THYM	2	2	0.500
